# Serum APE1 Autoantibodies: A Novel Potential Tumor Marker and Predictor of Chemotherapeutic Efficacy in Non-Small Cell Lung Cancer

**DOI:** 10.1371/journal.pone.0058001

**Published:** 2013-03-05

**Authors:** Nan Dai, Xiao-Jing Cao, Meng-Xia Li, Yi Qing, Ling Liao, Xian-Feng Lu, Shi-Heng Zhang, Zheng Li, Yu-Xin Yang, Dong Wang

**Affiliations:** 1 Cancer Center, Daping Hospital and Research Institute of Surgery, Third Military Medical University, Chongqing, China; 2 Department of Pathology, Chongqing Hospital of Traditional Chinese Medicine, Chongqing, China; Sapporo Medical University, Japan

## Abstract

Apurinic/apyrimidinic endonuclease 1 (APE1), which has the dual functions of both DNA repair and redox activity, has been reported to be highly expressed in non-small cell lung cancer (NSCLC), and this appears to be a characteristic related to chemotherapy resistance. In this study, we identified serum APE1 autoantibodies (APE1-AAbs) in NSCLC patients and healthy controls by immunoblotting and investigated the expression of APE1-AAbs by indirect ELISA from the serum of 292 NSCLC patients and 300 healthy controls. In addition, serum APE1-AAbs level alterations of 91 patients were monitored before and after chemotherapy. Our results showed that serum APE1-AAbs can be detected in both NSCLC patients and healthy controls. Serum APE1-AAbs were significantly higher than those of healthy controls and closely related to APE1 antigen levels both in tumor tissues and the peripheral blood. Moreover, the change in levels of serum APE1-AAbs in NSCLC is closely associated with the response to chemotherapy. These results suggest that APE1-AAbs is a potential tumor marker and predictor of therapeutic efficacy in NSCLC.

## Introduction

With its increasing incidence and mortality, lung cancer has become the largest cause of cancer deaths and a challenging clinical problem worldwide [1], [2]. Non-small cell lung cancer (NSCLC) is the main type of lung cancer, which is often diagnosed at an advanced stage so that patients have little prospect of effective and curative treatment, and this manifests with 5-year survival rates of <15% [3].

Screening for early NSCLC biomarkers, development of therapeutic efficacy predictors, and new drugs are vital factors in improving both the patients’ prognoses and survival [4], [5]. However, the present biomarkers and predictors for NSCLC still lack adequate sensitivity and specificity [6]. The carcinoembryonic antigen is one of the most widely-studied tumor markers in NSCLC, with an overall sensitivity of only approximately 40% [7]. Some new serum marker candidates such as vascular endothelial growth factor, stem cell factor, angiogenic cytokines, and hepatocyte growth factor/scatter factor may be potentially important, but most of them have not been established to be independent clinical prognostic indicators [8]. Therefore, more studies are required to discover novel biomarkers for assisting in the screening of NSCLC, which will greatly improve the therapeutic outcome of this malignant disease [9].

Circulating antibodies against tumor-associated antigens (TAAs) are a class of new serum biomarkers showing highly interesting properties, especially in early diagnosis for cancers. Autoantibodies are present in the serum of patients at an early stage when tumors cannot be clinically detected, even before TAAs can be detected [10], [11]. The persistence and stability of serum autoantibodies are primary advantages over other currently used serum biomarkers [12]. They show a longer lifetime (t_1/2_ between 7 and 30 days) in serum compared with TAAs, are highly stable in blood, and are not subject to proteolysis like other polypeptides [13], [14]. Moreover, as biochemically well-known molecules, antibodies can be detected by many available reagents and techniques both simply and cheaply. Over the past few years, an increasing articles have demonstrated that monitoring persons at increased risk of cancer for the presence of serum autoantibodies may allow early detection and/or prognosis in different cancers, including NSCLC [15]–[19]. Autoantibodies to p53, NY-ESO-1, survivin and CAGE have been shown to be diagnostic biomarkers for lung cancer patients [20]–[22].

Apurinic/apyrimidinic endonuclease 1 (APE1), which has the dual functions of both DNA repair and reduction-oxidation (redox) activity, is the major AP endonuclease of the base excision repair pathway. It plays an important role in the progression of NSCLC, as well as maintaining genome stability [23]. Elevated and ectopic expression of APE1 in tumor tissues is closely linked to poor prognosis and chemo- and radio-resistance in NSCLC [24]–[26]. Recently, Katsumata et al first identified APE1 autoantibodies (APE1-AAbs) in serum from systemic lupus erythematosus patients [27], but we have little knowledge about APE1-AAbs in NSCLC. We postulate that abnormally abundant or ectopic APE1 protein from tumor tissues may enter into serum and that APE1-AAbs might be detected in the peripheral blood of these NSCLC patients.

In this study, we detected APE1-AAbs using both immunoblotting and ELISA assay, then investigated the correlation among APE1-AAbs, serum APE1 antigen and the expression of APE1 protein in tumor tissues. Furthermore, we evaluated APE1-AAbs diagnostic value and the correlation with therapeutic efficacy in NSCLC patients. To our knowledge, this is the first report identifying serum APE1-AAbs in lung cancer.

## Materials and Methods

### Patients

This study was approved by the Ethics and Research Committee of the Daping faculty of Medicine, Third Military Medical University, China and written informed consent was obtained from all patients and healthy controls. Serum samples were obtained from 292 NSCLC patients and 300 healthy controls from January 2007 to June 2008. The demographic features and clinical characteristics of the studied groups are illustrated in Table 1. Ninety one patients who received two cycles of platinum-containing regimen were monitored before and after chemotherapy. The histopathological assessment was carried out separately by two pathologists, and then a consensus was made on discordant assessments. The staging system was carried out according to the 2003 AJCC classification. Therapeutic efficacy was defined by Response Evaluation Criteria in Solid Tumors, which classify the response into four categories: complete response (CR), partial response (PR), stable disease (SD) and progressive disease (PD). For data analysis, CR and PR were combined as responders who were sensitive to chemotherapy, while SD and PD were grouped as non-responders. Patients were excluded if they had immunologic disorders, evidence of acute or recent (<2 months) infection, recent major trauma, surgery, or treatment with immunosuppressive agents.

**Table 1:** Clinical characteristics and distribution of sera APE1-AAbs of studied groups.

Characteristics	NSCLC (N = 292)	Healthy (N = 300)	*p*
Age			
Range	40∼91	39∼86	
Mean	62	60	0.128
Gender			
Male	235(80.48%)	232(77.33%)	0.053
Female	57(19.52%)	68(22.67%)	
Smoking status			
Smoker	188(64.38%)	182(60.67%)	0.198
Non-smoker	104(35.62%)	118(39.33%)	

*p* values were calculated using chi-square test.

### Serum Collection

Peripheral blood samples were obtained from the healthy controls and from the patients after diagnosis but prior to any operations. For the 91 NSCLC patients treated with two cycle of platinum-contained chemotherapy, serum samples were obtained before chemotherapy and one month after chemotherapy. The whole blood samples were promptly centrifuged at 3000 rpm for 15 minutes and the supernatant stored at −80°C until use.

### Cell Culture

Human lung adenocarcinoma cells (A549) were purchased from American Type Culture Collection (Manassas, VA, USA) and cultured in RPMI 1640 (HyClone, Logan, UT, USA) supplemented with 10% fetal bovine serum, 100 units/mL penicillin and 100 µg/mL streptomycin. The cells were grown in 5% CO2 at 37°C.

### Dot Blot and Western Blot Analyses

The full-length APE1 fusion protein [with hexahistidine (His) tag, constructed and purified in our laboratory] was dotted onto NC membrane. The membrane was blocked for 2 h at 37°C with blocking buffer (5% BSA in TBST). The membrane was then incubated at 4°C overnight with serum from patients and healthy controls diluted 1∶500 in blocking buffer. After washing with TBST, the membrane was incubated for 1 h at 37°C with HRP-labeled goat anti-human IgG (1∶10000 dilution, Sigma, USA).

The expressions of serum APE1-Abbs were analyzed using Western blot analyses [27]. The protein extracted from A549 cells and APE1-His fusion protein was separated by 10% SDS–PAGE and then transferred to NC membrane. The membrane was blocked for 2 h at 37°C with blocking buffer. The membrane was then incubated at 4°C overnight with monoclonal anti-human APE1 antibody (1∶5000 dilution) and serum from lung cancer patients and healthy controls (1∶500 dilution). The membrane was incubated for 1 h at 37°C with HRP-labeled goat anti-mouse IgG (1∶10000 dilution) and HRP-labeled goat anti-human IgG (1∶10000 dilution).

### Enzyme-Linked Immunosorbent Assay (ELISA)

Indirect ELISA was used to detect serum APE1-AAbs and carried out as follows: (1) Microtiter plates (450∼500 ng/cm^2^, 8 well×12 strips, Costar Biosciences Inc, USA) were coated with 100 µl APE1-His fusion protein diluted with coating buffer (0.1 mol/L carbonate buffer, pH 9.6) at 1.0 µg/ml and incubated at 4°C overnight; (2) Plates were washed three times using washing buffer (0.05% PBST), 300 µl/well, then the non-specific sites in the wells were blocked with 200 µl blocking buffer (5% BSA in PBS) incubated for 2 h at 37°C; (3) After three washes, 100 µl/well of serum samples diluted 1∶300 were incubated for 1 h at 37°C, PBS was used as the calibration control; (4) The unbound compounds were washed away, 100 µl/well of HRP-labeled goat anti-human IgG (working concentration recommend 1∶50000 dilution) were incubated for 45 min at 37°C; (5) After washing six times, 100 µl/well of TMB (Pierce, USA) substrate solution was incubated for 10 min at 37°C; (6) The enzymatic reaction was stopped by 2 mol/L H_2_SO_4_ (50 µl/well) and then optical density (OD) was measured by microplate spectrophotometry at reference wavelength (450 nm). All samples were tested twice in two separate plates.

Improved sandwiching ELISA was used to detect serum APE1 antigen and carried out as follows: (1) Microtiter plates were coated with 100 µl mouse anti-human APE1 monoclonal antibody (1∶40000 dilution) incubated at 4°C overnight; (2) the wells were blocked for 2 h at 37°C; (3) 100 µl/well of serum samples were incubated for 1 h at 37°C, PBS was used as the calibration control; (4) 100 µl rabbit anti-human APE1 polyclonal antibody (1∶5000 dilution) incubated for 1 h at 37°C; (5) 100 µl/well of HRP-labeled goat anti-rabbit IgG (1∶5000 dilution) were incubated for 45 min at 37°C; (6) After washing six times, 100 µl/well of TMB substrate solution was incubated for 10 min at 37°C and stopped by 2 mol/L H_2_SO_4_, optical density (OD) was measured by microplate spectrophotometry at reference wavelength (450 nm). All samples were tested twice in two separate plates.

### APE1 Immunohistochemistry and Scoring

The expression of APE1 protein of NSCLC patients were analyzed using immunohistochemistry. Slides were cut to 4 µm sections and incubated with mouse anti-human APE1 monoclonal antibody (1∶2000 dilution). Each specimen’s histologic diagnosis was confirmed by a pathologist as previously studies with APE1 [26]. Scored by the percentage of cell staining and intensity of staining as previous studies [28], [29], tissues were classified into four categories: 0, 1, 2 and 3 corresponding to negative, weak, moderate and strong expression. The score 0 and score 1 were considered low expression, while score 2 and score 3 were considered high expression.

### Statistical Analyses

The data were expressed as median or mean ± standard deviation (SD). Associations between clinical characteristics and APE1 autoantibodies or antigen were assessed by independent *t*-test, chi-square test and one-way analysis of variance. The association was assessed by Receiver operating characteristic (ROC) curve was used to evaluate the sensitivity and specificity. Correlation strength was assessed by the Spearman correlation test. Associations between the levels APE1-AAbs of pre- and post-chemotherapy were analyzed by paired *t*-test. In all calculations, *p*<0.05 was regarded as statistically significant. All statistical procedures were performed by using GraphPad Prism software, version 5.0 and SPSS software, version 13.0 for Windows.

## Results

### Identification of Serum APE1-AAbs in Healthy Controls and NSCLC Patients

**Figure 1:**
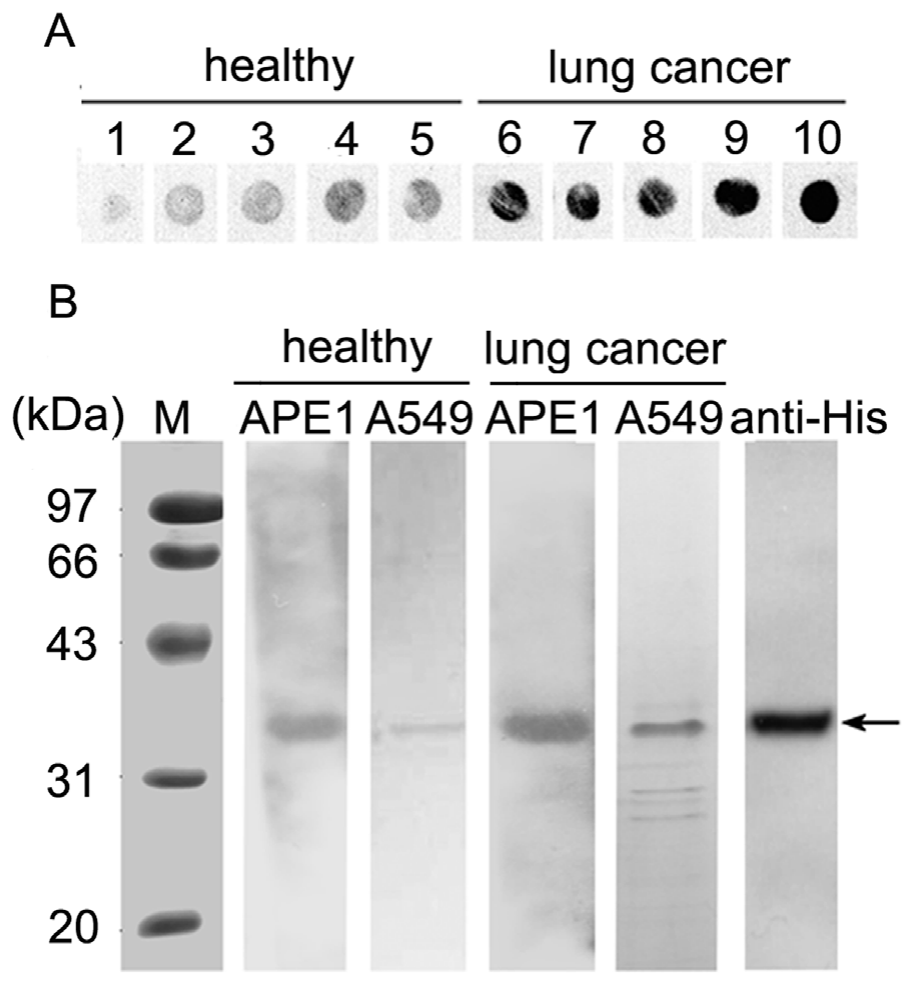
Dot blot and Western blot for identifying serum APE1-AAbs. A, Representative results of detection of sera APE1-AAbs by dot blot assay. Lanes 1∼5: serum of healthy subjects. Lanes 6∼10: serum of NSCLC patients. B, Serum APE1-AAbs of healthy and NSCLC patients were detected by Western blot analysis. Lane M, protein molecular weight markers; APE1, APE1 fusion protein with His tag; A549, total cell protein extracted from A549 cells; Anti-His, APE1 fusion protein detected by His antibody.

Using serum samples for dot blot and Western bolt analyses, we found that in both NSCLC patients and healthy controls, autoantibodies in the serum recognized purified APE1-His fusion protein and APE1 protein in A549 whole cell lyses (Fig. 1A and 1B). In addition, the immunoreactive dot and band signals from NSCLC patients were stronger than the healthy controls. These results suggested that APE1-AAbs can be detected in the serum from both NSCLC patients and healthy controls.

### The Distribution of APE1-AAbs in Serum of Healthy Controls and NSCLC Patients

**Figure 2:**
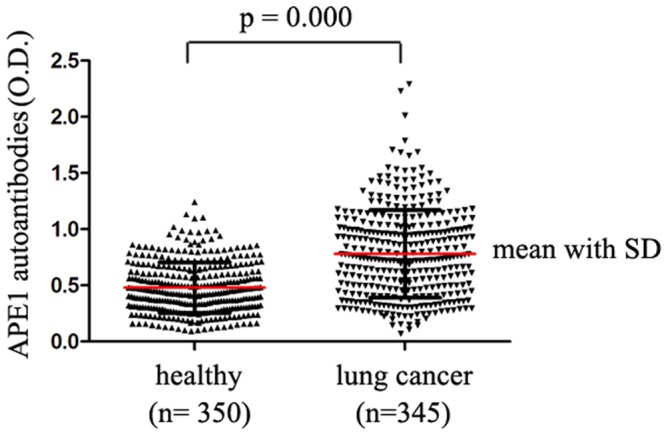
Serum APE1-AAbs level in NSCLC patients and healthy subjects assessed by ELISA. Among 292 NSCLC patients, the mean with SD of APE1-AAbs concentration was 0.79±0.40 (OD_450_), which was significantly higher than 0.47±0.22 (OD_450_) in healthy volunteers (*p* = 0.000, *t*-test).

According to the demographic features and clinical characteristics of NSCLC patients and healthy controls (described in Table 1), there was no statistical difference in the distribution of age, gender and smoking status between the two groups. Indirect ELISA method was built (described in Fig. S1) and used to detect the prevalence of serum autoantibodies against APE1. Among 292 NSCLC patients, the mean with SD of APE1-AAbs concentration was 0.79±0.40 (OD_450_), which was significantly higher than 0.47±0.22 (OD_450_) in healthy controls (*p* = 0.000, *t*-test) (Fig. 2). The levels of APE1-AAbs showed a normal distribution.

We also investigated the relationship between APE1-AAbs and the clinical characteristics. Among 300 healthy controls, the mean with SD of APE1-AAbs concentration of healthy smokers was 0.47±0.01 (OD_450_), which was no significant difference with the non-smokers of healthy controls (0.48±0.003, OD_450_) (*p* = 0.679, *t*-test). These results suggested that the serum APE1-AAbs levels seemed not to respond to smoking status. Furthermore, the results revealed that serum APE1-AAbs of NSCLC patients did not correlate with clinical parameters like gender, different TNM stages and histopathological types, including smoking status, as shown in Table 2 (*p*>0.05). Although patients at stage IV had a higher APE1-AAbs positive rate (71/172, 41.28%) than at stage III (27/81, 33.33%),the difference was not statistically significant ((*p*>0.05).

**Table 2:** Association of serum APE1-AAbs level with clinical characteristics among NSCLC groups.

Patients Characteristics	N (%)	APE1-AAbs level(OD_450_)	*p*		Positive N (%)	*p*
Age						
<60	135	0.78±0.39	0.905	55 (40.74%)		0.506
≥60	157	0.78±0.40		58 (36.94%)		
Gender						
Male	235(80.48%)	0.80±0.38	0.354	93(39.57%)		0.650
Female	57(19.52%)	0.74±0.45		20(35.09%)		
Smoking status						
Smoker	188(64.38%)	0.80±0.39	0.445	75(39.89%)		0.617
Non-smoker	104(35.62%)	0.76±0.41		38(36.54%)		
Histological types						
Adeno	116(39.73%)	0.80±0.39	*0.505	45(38.79%)		*0.805
Squamous	166(56.85%)	0.77±0.41		62(37.35%)		
LCLC	2(0.68%)	0.62±0.01		0(0.00%)		
Others	8(2.74%)	0.92±0.13		6(75.00%)		
TNM stage						
I+II	39(13.36%)	0.72±0.37	^a^0.436	15(39.32%)		^a^0.683
III	81(27.74%)	0.78±0.37	^b^0.626	27(33.332%)		^b^0.269
IV	172(58.90%)	0.80±0.41	^c^0.221	71(41.28%)		^c^0.857
Total	292 (100%)	0.79±0.40		113(38.70%)		

Adeno: adenocarcinoma; LCLC: large cell lung cancer. Independent *t*-test and ANOVA were used to analyze associations between the APE1-AAbs level (OD) and clinical characteristics. Chi-square test was used to analyze associations between the APE1-AAbs positive and clinical characteristics.

* Adeno versus Squamous; a: I+II versus III; b: III versus IV; c: I+II versus IV.

### The Diagnostic Value of APE1-AAbs in NSCLC

We further explored the potential diagnostic value of APE1-AAbs in NSCLC. Based on the principle of cutoff value [30], the cutoff level of APE1-AAbs was calculated as mean+2SD (0.47+2×0.22 = 0.91) of 300 serum samples from healthy controls. Thus, 113 (38.70%) NSCLC patients, versus 8 (2.67%) healthy controls, were defined as APE1-AAbs positive, which indicated that the difference between the two groups was significant (*p* = 0.000, *chi-square* test).

**Figure 3:**
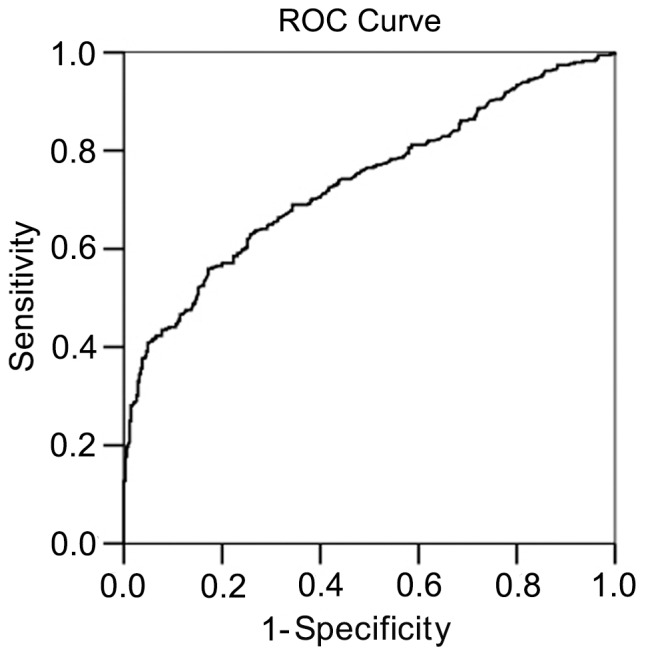
Receiver operating characteristic curves for APE1-AAbs level for NSCLC detection. Serum concentrations of APE1-AAbs levels among 292 NSCLC patients and 300 healthy controls were determined by ELISA. The diagnostic potentials of APE1-AAbs were assessed by ROC curves. The AUC value was 0.745.

The performance of all serum samples was summarized with an ROC curve. The predictive performance of APE1-AAbs level was determined by plotting sensitivity (true positive) against 1-specificity (false positive) values. For each possible cut-point, the resulting sensitivity and specificity were indicated as a point on the graph. The area underneath the curve (AUC) was 0.745 (Fig. 3), suggesting that the APE1-AAbs was meaningful as a potential diagnostic biomarker.

### Correlation Analysis among APE1-AAbs, Serum APE1 Antigen and APE1 Protein Expression in NSCLC Tissues

**Figure 4:**
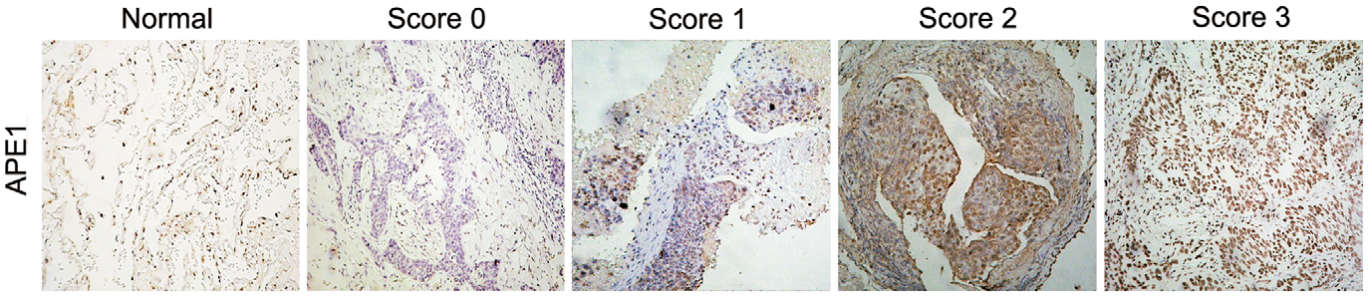
APE1 protein expression in NSCLC tissues. Representative APE1 immunostaining of NSCLC tissues. APE1 staining was observed three subcellular locations in tumor tissues: the nucleus, the cytoplasm and both in nucleus and cytoplasm. Samples were scored as follows: 0, absence or positive cell percentage less than 25%; 1, positive cell percentage between 25% and 50%; 2, positive cell percentage between 50% and 75%; 3, positive cell percentage more than75%.

Considering that for the APE1-AAbs response to occur, the APE1 antigen needs to be detected to show their relationship. A total of 42 NSCLC tissues were investigated the expression of APE1 protein using immunohistochemistry. APE1 staining was observed three subcellular locations in tumor tissues: the nucleus (8/42, 19.05%), the cytoplasm (3/42, 7.14%) and both in nucleus and cytoplasm (31/42, 73.81%) as shown in Fig. 4 and Table S1. The serum APE1-AAbs levels of nucleus expression group were no statistical difference with the ectopic expression group (including cytoplasm expresssion and nucleus/cytoplasm coexpression) (*p* = 0.610, shown in Table S1). Six NSCLC cases (15%) showed strong APE1 expression, 20 cases (50%) and 12 cases (30%) showed moderate and weak expression, respectively. The high APE1 expression (score 2 and 3) and low expression (score 0 and 1) in NSCLC tissues were shown no significant difference among age, gender, smoking status, histological type and TMN stages (*p*>0.05, *chi-square* test, shown in Table S2), which were the same as the previously reviews [24], [31]. Interestingly, a positive correlation between serum APE1-AAbs and APE1 protein expression in NSCLC tissues was found, being statistically significant (*p*<0.001, Spearman) and with the correlation coefficient >0.50, as shown in Table S1.

Furthermore, we analyzed the serum APE1 antigen in 137 NSCLC patients who had been tested for APE1-AAbs using improved sandwiching ELISA assay. The mean with SD of serum APE1 antigen concentration was 0.73±0.41 (OD_450_), and there was no significant difference between serum APE1 antigen and clinical characteristics (*p*>0.05, shown in Table S3). As expected, we found APE1 antigen and APE1-AAbs in peripheral blood were also positively correlated, with the correlation coefficient being >0.50 and statistically significant (p<0.001, Spearman). These findings suggested that the expressions of APE1 autoantibodies were closely related to the APE1 antigen levels.

### Increase of Serum APE1-AAbs between Pre- and Post-chemotherapy is Associated with Therapeutic Efficacy

**Figure 5:**
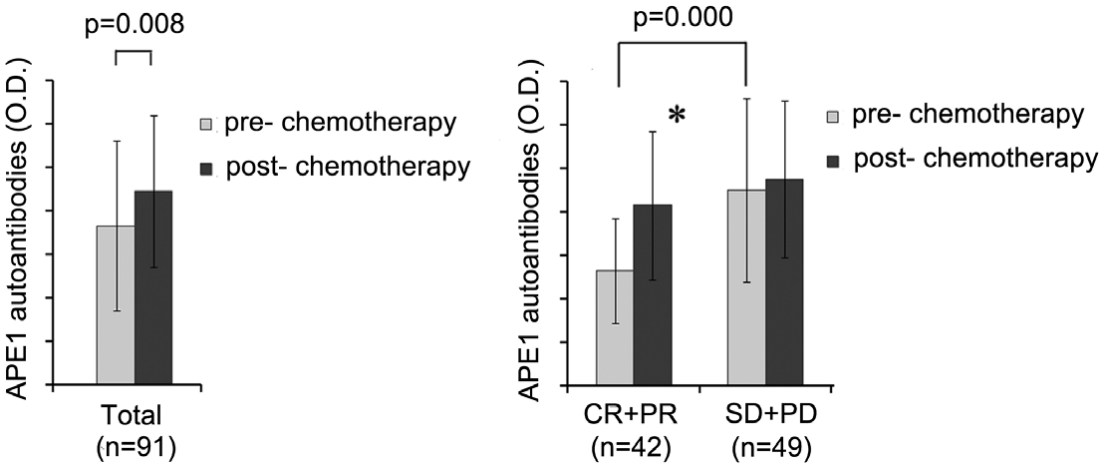
Distribution of serum APE1-AAbs before and after chemotherapy. The serum APE1-AAbs level was much higher in the poor chemotherapeutic response group than in the good response group (*p* = 0.000). There was a significant difference in the group with good platinum-based chemotherapeutic response before and after chemotherapy (*p*<0.001), while there was no difference in the group with poor platinum-based chemotherapeutic response before and after chemotherapy (*p* = 0.393). *before chemotherapy versus after chemotherapy (*p*<0.001). *p* value were obtained after comparing the levels APE1-AAbs of pre- and post- chemotherapy, as determined by paired *t*-test.

To determine the correlation between serum APE1-AAbs level and therapeutic response, the APE1-AAbs levels between pre- and post-chemotherapy were analyzed in 91 NSCLC patients who received 2 cycles of platinum-based regimen. Generally, serum APE1-AAbs level significantly increased after chemotherapy (*p* = 0.008) (Fig. 5A). Among 91 patients, 11 (12.09%) patients achieved PD, 38 (41.76%) patients achieved SD, 30 (32.97%) patients achieved PR, and 12 (13.19%) patients achieved CR. Pre-chemotherapy serum APE1-AAbs of patients who were sensitive to chemotherapy were significantly lower than that of non-responders (*p* = 0.000) (Fig. 5B). Serum APE1-AAbs of responders after chemotherapy were significantly increased (*p* = 0.000), while APE1-AAbs of non-responders did not change significantly (*p* = 0.393), as shown in Fig. 5 and Table 3.

**Table 3:** Characteristics and serum APE1-AAbs distribution of NSCLC patients with pre- and post- chemotherapy.

Therapeutic efficacy	N	Age(mean)	Gender (N)		Smoking status (N)		Histological types (N)			TNM stage (N)			APE1-AAbs		*p*
			M	F	Smoker	Non smoker	Adeno	Squamous	Others	I+II	III	IV	pre- chemotherapy (OD)	post- chemotherapy (OD)	
CR+PR	42	60.17	37	5	42	0	18	24	0	15	13	14	0.53±0.24	0.83±0.34	0.000
SD+PD	49	65.10	41	8	36	13	15	34	3	10	15	24	0.90±0.42	0.95±0.36	0.393
Total	91	62.82	78	13	78	13	33	58	3	25	28	38	0.73±0.39	0.89±0.35	0.008

*p* value were obtained after comparing the levels APE1-AAbs of pre- and post- chemotherapy, as determined by paired *t*-test.

## Discussion

Experimental studies have shown that autoantibodies are potential biomarkers for early cancer diagnosis and predictors for treatment response. However, because autoantibodies are part of the normal immune response, candidate autoantibodies for early cancer detection and prognosis of cancers should be against oncogenic-related antigens. In this regard, autoantibodies to DNA repair proteins are promising candidates. DNA repair proteins play critical roles not only in maintaining genomic stability but also in the progression of lung cancer [32], [33]. Both antigens and autoantibodies to antigens involved in DNA repair pathways, such as p53 [34]–[36] and Ku [37]–[39], have been highlighted as factors involved in tumorigenesis and as biomarkers in lung cancer, breast cancer, and leukemia.

As one of the DNA repair proteins, APE1 also plays an important role in cell survival, and its high expression is correlated with tumor characteristics [40], [41]. Having the dual functions of both DNA repair and redox activity, it is not only responsible for repairing DNA AP sites caused by oxidative and alkylation damage in order to maintain genomic integrity [42], but also functions as a redox factor regulating the DNA binding activity of transcription factors such as p53, NF-κB and AP-1 that play crucial roles in suppression of carcinogenesis and tumor progression [43], [44]. Using RT-PCR and immunohistochemical staining, previous studies have shown that changes in APE1 expression levels and/or patterns in NSCLC tissues and peripheral blood occurred in the early period of tumorigenesis and were closely related to tumor development, progress, and unfavorable prognosis [31], [45], [46]. These studies suggested that APE1 antigen could be a candidate for cancer screening, early auxiliary diagnosis, and prognostic and predictive evaluation in many cancer tissues including NSCLC [47], [48]. The present study validates an assay for the detection of APE1 autoantibodies both in the peripheral blood of NSCLC patients as well as healthy controls. Using immunoblotting and ELISA assay analysis, we showed that APE1 is a specific autoimmune antigen that stimulates organisms to generate anti-APE1 antibodies. Statistically significant associations between APE1-AAbs and NSCLC are demonstrated for the first time.

The mechanism of how the APE1 protein triggers the immune response is still not entirely clear. We presume the possible mechanism is: the majority of tumor tissues have APE1 over-expression and translocation. The tumor cell multiplication is excessively quick. With spontaneously continual cell apoptosis and necrosis, a large number of cellular proteins are released into the blood and can not be timely removed. The immune system senses the cellular proteins which are overexpressed in carcinogenesis, cytoplasmic translocalization, disregulated stabilization, and mutation or altered degradation and subsequently produces autoantibodies in response to the presence of these aberrant antigens [49]. Thus, we tried to verify whether various APE1 expression location could contribute to APE1-AAbs generation, but the result showed that the levels of serum APE1-AAbs between the nucleus expression group and the ectopic expression group was no difference (*p* = 0.610). This result should be investigated in a bigger sample size in future. Considering that tumor burden might induce a high level of APE1-AAbs, we will also investigate the APE1-AAbs before and post surgery in our further studies to proving this hypothesis.

In our study, the presence of APE1-AAbs showed the possibility of use as a biomarker for NSCLC. The finding of APE1-AAbs expression in the serum of 38.70% (113/292) of NSCLC patients was statistically significantly higher than that of healthy controls (2.67%, 8/300) (*p* = 0.000). The value of the area under the ROC curve of APE1-AAbs was 0.745, meaningful for a predictive model, because in ELISA detection, an AUC value of 0.7∼0.9 (70%∼90%) indicates moderate association between prediction and true outcome [50]. Moreover, the APE1-AAbs proved to correlate well with APE1 antigen levels both in NSCLC tissues and peripheral blood in the present study. We noted that the correlation between APE1 mRNA expression in NSCLC tissues, normal tissues and blood had been demonstrated to be significantly correlated, which suggested that measurement of mRNA levels of APE1 in peripheral blood samples could instead of tissue samples to determine prognostic and predictive factors in NSCLC [31]. Accordingly, we could allow to test simply with APE1-AAbs in peripheral blood samples instead of tissue samples to determine diagnosis and prognostic factors in NSCLC patients.

However, although promising, these novel findings require further investigation and careful interpretation in order to reach decisive conclusions because the difference of serum APE1-AAbs levels between NSCLC and healthy controls was not very high. The variations of APE1-AAbs in the study population are extremely high, though the levels of serum APE1-AAbs showed a normal distribution. The reasons for these are unclear, but may be related to patient selection, differences in epitope detection, or the limitations of the array and the nature of autoantibody responses in cancer. It is worth mentioning that APE1-AAbs in various cancers, including more extensive samples of lung cancer, need to be verified in order to establish the clinical significance of anti-APE1 antibodies. Further studies on whether APE1-AAbs have diagnostic efficacy or can be usefully combined with other tumor markers will be summarized in our next study.

Histology is a basic diagnostic indicator in NSCLC patients [51]. As a phenotype, it can be more reproducible and consistent compared to information on smoking habits, especially in this kind of retrospective study [23]. Mitsudomi *et al* reported that p53 autoantibodies were significantly more prevalent in patients with squamous cell carcinoma (27%) than in those with adenocarcinoma (15%) (*p*  = 0.05), while there was also a statistically significant difference in the incidence of p53 autoantibodies between the early disease group (stage I and II) (14%) and the advanced disease group (stage IIIa∼IV, 30%) (*p = 0*.0079) [52]. Villin1 and CK18 autoantibodies are considered to be useful markers in lung adenocarcinoma [53]. Therefore, we investigated the relationship between levels of APE1-AAbs and lung cancer histotype. We found that the level of serum APE1-AAbs did not correlate with histopathological types (squamous cell carcinoma or adenocarcinoma) and different TNM stages or clinical parameters such as gender and smoking status. These data were similar to previous studies of APE1 protein in lung cancer tissues [31], [54].

High expression of APE1 protein has been reported to closely associate with cisplatin resistance in ovarian cancer [29], head and neck squamous cell cancer [31], and NSCLC [26]. The data in this manuscript further confirm this relationship between APE1 and platinum-based chemoresistance. Before chemotherapy, we observed that the APE1-AAbs levels of the CR+PR groups which were sensitive to platinum-based treatment were significantly lower than that of the SD+PD groups which were resistant to chemotherapy (*p* = 0.000). Moreover, APE1-AAbs levels were significantly increased after chemotherapy, especially in the positive response group (*p* = 0.000). To date, the mechanism of APE1 involved in resistance to platinum-based chemotherapy remains unclear. Chattopadhyay *et al* showed that acetylated APE1 could stably interact with Y-box-binding protein 1 and enhance its binding to the Y-box element leading to the activation of the multidrug resistance gene MDR1 [55]. Wu *et al* reported that cytoplasmic APE1 could enhance lung tumor malignancy through NF-κB activation, suggesting that the combination of cisplatin with specific redox inhibitors could improve chemotherapeutic response [56]. We presume that platinum reagents can kill lung cancer cells, induce cellular necrosis, release APE1 protein from the tumor burden to the extracellular environment and circulation, and subsequently trigger the immune response to develop APE1-AAbs. Theoretically, chemotherapy resistant patients will have less tumor cells death after treatment, release less APE1 antigen into the blood and induce to a smaller increase of serum APE1-AAbs. On the contrary, the patients who are sensitive to platinum-based chemotherapy should contain more APE1-AAbs in their peripheral blood after treatment. Moreover, before chemotherapy, the patients have more APE1-AAbs in their peripheral blood indicating that more APE1 protein is present in tumor tissues compared to normal tissue, thereby leading to resistance to platinum-based treatment. Our data just verified these points. Therefore, we believe the APE1-AAbs can be used to predict the chemotherapy response in NSCLC before and after platinum-based treatment.

In conclusion, we identified that autoantibodies against APE1 protein was present in serum both from lung cancer patients and healthy controls. The level of APE1-AAbs in lung cancer patients was significantly higher than healthy controls. A statistically significant correlation was found between APE1-AAbs and APE1 antigen both in NSCLC tissues and peripheral blood. Serum APE1-AAbs in the positive chemotherapeutic response group was significantly higher after chemotherapy. The results not only further support that APE1 expression is closely related to lung cancer and chemotherapy response, but also provide clues that APE1-AAbs possess the potential to be a novel serum diagnostic and predictive marker for lung cancer. Moreover, further work is underway to both replicate these findings in a study with a larger cohort in lung cancer and other malignant disease.

## Supporting Information

Figure S1 **Optimal Serum dilution for ELISA technique.** Optimal dilution was determined by titration technique. Dilution experiments showed that the APE1-AAbs titer differed from one patient to another. Two samples (p083487, p084393) from healthy controls displayed a low titer of APE1-AAbs while another two samples (p080195, p080643) from NSCLC patients had a high titer of APE1-AAbs. P080195 sample displayed a positive signal until diluted to 1∶1000, whereas p080643 showed a strong signal for a dilution of 1∶2000. These serum samples could be diluted 1∶1000 and still yield a positive signal. The optimal dilution of serum we selected was 1∶300.
(TIF)

Table S1 **Association between APE1 protein expression and serum APE1-AAbs in 42 NSCLC patients.** *was correlation coefficient. The correlation of serum APE1-AAbs levels between nucleus expression group and ectopic expression group (cytoplasm expresssion and nucleus/cytoplasm coexpression) was statistically analyzed by Mann-Whitney.
(DOCX)

Table S2 **Association between clinical characteristics and APE1 protein expression in NSCLC tissues.** *p* values were calculated using chi-square test.
(DOCX)

Table S3 **Association between serum APE1 antigen and serum APE1-AAbs in 137 NSCLC patients.** *was correlation coefficient.
(DOCX)

## References

[1] Jemal A, Bray F (2011) Center MM, Ferlay J, Ward E, et al (2011) Global cancer statistics. CA Cancer J Clin 61: 69–90. 21296855 10.3322/caac.20107

[2] Wang JB, Jiang Y, Wei WQ, Yang GH, Qiao YL, et al (2010) Estimation of cancer incidence and mortality attributable to smoking in China. Cancer Causes Control 21: 959–965. 20217210 10.1007/s10552-010-9523-8

[3] Jemal A, Siegel R, Xu J, Ward E (2010) Cancer statistics, 2010. CA Cancer J Clin 60: 277–300. 20610543 10.3322/caac.20073

[4] Postmus PE (2008) Screening for lung cancer, an ongoing debate. Ann Oncol 19 Suppl 7 vii25–27. 10.1093/annonc/mdn464 18790961

[5] Konopa K (2010) Do we have markers to select patients for adjuvant therapies of non-small-cell lung cancer? Ann Oncol 21 Suppl 7 vii199–202. 20943615 10.1093/annonc/mdq452

[6] Indovina P, Marcelli E, Maranta P, Tarro G (2011) Lung cancer proteomics: recent advances in biomarker discovery. Int J Proteomics 2011: 726869. 22229091 10.1155/2011/726869 PMC3196861

[7] Tufman A, Huber RM (2010) Biological markers in lung cancer: A clinician’s perspective. Cancer Biomark 6: 123–135. 20660959 10.3233/CBM-2009-0124 PMC12922859

[8] Bharti A, Ma PC, Salgia R (2007) Biomarker discovery in lung cancer–promises and challenges of clinical proteomics. Mass Spectrom Rev 26: 451–466. 17407130 10.1002/mas.20125

[9] Ostroff RM, Bigbee WL, Franklin W, Gold L, Mehan M, et al (2010) Unlocking biomarker discovery: large scale application of aptamer proteomic technology for early detection of lung cancer. PLoS One 5: e15003. 21170350 10.1371/journal.pone.0015003 PMC2999620

[10] Desmetz C, Mange A, Maudelonde T, Solassol J (2011) Autoantibody signatures: progress and perspectives for early cancer detection. J Cell Mol Med 15: 2013–2024. 21651719 10.1111/j.1582-4934.2011.01355.x PMC4394213

[11] Folli F, Solimena M, Cofiell R, Austoni M, Tallini G, et al (1993) Autoantibodies to a 128-kd synaptic protein in three women with the stiff-man syndrome and breast cancer. N Engl J Med 328: 546–551. 8381208 10.1056/NEJM199302253280805

[12] Tan HT, Low J, Lim SG, Chung MC (2009) Serum autoantibodies as biomarkers for early cancer detection. FEBS J 276: 6880–6904. 19860826 10.1111/j.1742-4658.2009.07396.x

[13] Kobold S, Lutkens T, Cao Y, Bokemeyer C, Atanackovic D (2010) Autoantibodies against tumor-related antigens: incidence and biologic significance. Hum Immunol 71: 643–651. 20433885 10.1016/j.humimm.2010.03.015

[14] Atassi MZ, Casali P (2008) Molecular mechanisms of autoimmunity. Autoimmunity 41: 123–132. 18324481 10.1080/08916930801929021

[15] Nesterova M, Johnson N, Cheadle C, Cho-Chung YS (2006) Autoantibody biomarker opens a new gateway for cancer diagnosis. Biochim Biophys Acta 1762: 398–403. 16483750 10.1016/j.bbadis.2005.12.010

[16] Iizasa T, Fujisawa T, Saitoh Y, Hiroshima K, Ohwada H (1998) Serum anti-p53 autoantibodies in primary resected non-small-cell lung carcinoma. Cancer Immunol Immunother 46: 345–349. 9756419 10.1007/s002620050496 PMC11037331

[17] Saleh J, Brunner C, Golzer R, Nastainczyk W, Montenarh M (2006) p53 autoantibodies from patients with head and neck cancer recognise common epitopes on the polypeptide chain of p53. Cancer Lett 233: 48–56. 15913883 10.1016/j.canlet.2005.02.040

[18] Macdonald IK, Allen J, Murray A, Parsy-Kowalska CB, Healey GF, et al (2012) Development and validation of a high throughput system for discovery of antigens for autoantibody detection. PLoS One 7: e40759. 22815807 10.1371/journal.pone.0040759 PMC3399887

[19] Milne K, Barnes RO, Girardin A, Mawer MA, Nesslinger NJ, et al (2008) Tumor-infiltrating T cells correlate with NY-ESO-1-specific autoantibodies in ovarian cancer. PLoS One 3: e3409. 18923710 10.1371/journal.pone.0003409 PMC2561074

[20] Boyle P, Chapman CJ, Holdenrieder S, Murray A, Robertson C, et al (2011) Clinical validation of an autoantibody test for lung cancer. Ann Oncol 22: 383–389. 20675559 10.1093/annonc/mdq361 PMC3030465

[21] Tureci O, Mack U, Luxemburger U, Heinen H, Krummenauer F, et al (2006) Humoral immune responses of lung cancer patients against tumor antigen NY-ESO-1. Cancer Lett 236: 64–71. 15992994 10.1016/j.canlet.2005.05.008

[22] Karanikas V, Khalil S, Kerenidi T, Gourgoulianis KI, Germenis AE (2009) Anti-survivin antibody responses in lung cancer. Cancer Lett 282: 159–166. 19380192 10.1016/j.canlet.2009.03.015

[23] Evans AR, Limp-Foster M, Kelley MR (2000) Going APE over ref-1. Mutat Res 461: 83–108. 11018583 10.1016/s0921-8777(00)00046-x

[24] Zhang YS, Fan S, Wang D, Yang Z, Xiang D (2007) APE1 expression and its correlation with prognosis in non small cell lung cancer. Acta Academiae Medicinae Militaris Tertiae 29: 776–778.

[25] Yoo DG, Song YJ, Cho EJ, Lee SK, Park JB, et al (2008) Alteration of APE1/ref-1 expression in non-small cell lung cancer: the implications of impaired extracellular superoxide dismutase and catalase antioxidant systems. Lung Cancer 60: 277–284. 18061304 10.1016/j.lungcan.2007.10.015

[26] Wang D, Xiang DB, Yang XQ, Chen LS, Li MX, et al (2009) APE1 overexpression is associated with cisplatin resistance in non-small cell lung cancer and targeted inhibition of APE1 enhances the activity of cisplatin in A549 cells. Lung Cancer 66: 298–304. 19324449 10.1016/j.lungcan.2009.02.019

[27] Katsumata Y, Kawaguchi Y, Baba S (2011) Identification of Three New Autoantibodies Associated with Systemic Lupus Erythematosus Using Two Proteomic Approaches. Molecular & Cellular Proteomics 10. 10.1074/mcp.M110.005330 PMC3108835 21474795

[28] Wang D, Luo M, Kelley MR (2004) Human apurinic endonuclease 1 (APE1) expression and prognostic significance in osteosarcoma: enhanced sensitivity of osteosarcoma to DNA damaging agents using silencing RNA APE1 expression inhibition. Mol Cancer Ther 3: 679–686. 15210853

[29] Zhang Y, Wang J, Xiang D, Wang D, Xin X (2009) Alterations in the expression of the apurinic/apyrimidinic endonuclease-1/redox factor-1 (APE1/Ref-1) in human ovarian cancer and indentification of the therapeutic potential of APE1/Ref-1 inhibitor. Int J Oncol 35: 1069–1079. 19787261

[30] Lubin R, Schlichtholz B, Teillaud JL, Garay E, Bussel A, et al (1995) p53 Antibodies in Patients with Various Types of Cancer: Assay, Identification, and Chatacterization. Clin Cancer Res 1: 1463–1469. 9815945

[31] Schena M, Guarrera S, Buffoni L, Salvadori A, Voglino F, et al.. (2012) DNA repair gene expression level in peripheral blood and tumour tissue from non-small cell lung cancer and head and neck squamous cell cancer patients. DNA Repair (Amst). 10.1016/j.dnarep.2012.01.003 22284908

[32] Nuciforo PG, Luise C, Capra M, Pelosi G, d’Adda di Fagagna F (2007) Complex engagement of DNA damage response pathways in human cancer and in lung tumor progression. Carcinogenesis 28: 2082–2088. 17522062 10.1093/carcin/bgm108

[33] Gorgoulis VG, Vassiliou LV, Karakaidos P, Zacharatos P, Kotsinas A, et al (2005) Activation of the DNA damage checkpoint and genomic instability in human precancerous lesions. Nature 434: 907–913. 15829965 10.1038/nature03485

[34] Chapman CJ, Thorpe AJ, Murray A, Parsy-Kowalska CB, Allen J, et al (2011) Immunobiomarkers in small cell lung cancer: potential early cancer signals. Clin Cancer Res 17: 1474–1480. 21138858 10.1158/1078-0432.CCR-10-1363

[35] Neri M, Betta P, Marroni P, Filiberti R, Cafferata M, et al (2003) Serum anti-p53 autoantibodies in pleural malignant mesothelioma, lung cancer and non-neoplastic lung diseases. Lung Cancer 39: 165–172. 12581569 10.1016/s0169-5002(02)00449-x

[36] Messmer BT, Nour-Omid TS, Ghia E, Sanchez AB, Kipps TJ (2011) Autoantibodies against p53 are associated with chromosome 17p deletions in chronic lymphocytic leukemia. Leuk Res 35: 965–967. 21570119 10.1016/j.leukres.2011.04.009 PMC3116639

[37] Qiu J, Choi G, Li L, Wang H, Pitteri SJ, et al (2008) Occurrence of autoantibodies to annexin I, 14–3-3 theta and LAMR1 in prediagnostic lung cancer sera. J Clin Oncol 26: 5060–5066. 18794547 10.1200/JCO.2008.16.2388 PMC2652098

[38] Gullo C, Au M, Feng G, Teoh G (2006) The biology of Ku and its potential oncogenic role in cancer. Biochim Biophys Acta 1765: 223–234. 16480833 10.1016/j.bbcan.2006.01.001

[39] Fernandez Madrid F (2005) Autoantibodies in breast cancer sera: candidate biomarkers and reporters of tumorigenesis. Cancer Lett 230: 187–198. 16297705 10.1016/j.canlet.2004.12.017

[40] Tell G, Damante G, Caldwell D, Kelley MR (2005) The Intracellular Localization of APE1/Ref-1:More than a Passive Phenomenon? Antioxid Redox Signal 7: 365–384. 10.1089/ars.2005.7.367 15706084

[41] Tell G, Quadrifoglio F, Tiribelli C, Kelley MR (2009) The many functions of APE1/Ref-1: not only a DNA repair enzyme. Antioxid Redox Signal 11: 601–620. 18976116 10.1089/ars.2008.2194 PMC2811080

[42] Hegde ML, Hazra TK, Mitra S (2008) Early steps in the DNA base excision/single-strand interruption repair pathway in mammalian cells. Cell Res 18: 27–47. 18166975 10.1038/cr.2008.8 PMC2692221

[43] Sweasy JB, Lang T, DiMaio D (2006) Is base excision repair a tumor suppressor mechanism? Cell Cycle 5: 250–259. 16418580 10.4161/cc.5.3.2414

[44] Bhakat KK, Mantha AK, Mitra S (2009) Transcriptional regulatory functions of mammalian AP-endonuclease (APE1/Ref-1), an essential multifunctional protein. Antioxid Redox Signal 11: 621–638. 18715144 10.1089/ars.2008.2198 PMC2933571

[45] Singh KK, Kulawiec M, Still I, Desouki MM, Geradts J, et al (2005) Inter-genomic cross talk between mitochondria and the nucleus plays an important role in tumorigenesis. Gene 354: 140–146. 15979824 10.1016/j.gene.2005.03.027

[46] Abbotts R, Madhusudan S (2010) Human AP endonuclease 1 (APE1): from mechanistic insights to druggable target in cancer. Cancer Treat Rev 36: 425–435. 20056333 10.1016/j.ctrv.2009.12.006

[47] Luo M, Kelley MR (2004) Inhibition of the human apurinic/apyrimidinic endonuclease (APE1) repair activity and sensitization of breast cancer cells to DNA alkylating agents with lucanthone. Anticancer Res 24: 2127–2134. 15330152

[48] Fishel ML, Kelley MR (2007) The DNA base excision repair protein Ape1/Ref-1 as a therapeutic and chemopreventive target. Mol Aspects Med 28: 375–395. 17560642 10.1016/j.mam.2007.04.005

[49] Zinkernagel RM (2000) What is missing in immunology to understand immunity? Nat Immunol 1: 181–185. 10973269 10.1038/79712

[50] Fan J, Upadhye S, Worster A (2006) Understanding receiver operating characteristic (ROC) curves. CJEM 8: 19–20. 17175625 10.1017/s1481803500013336

[51] Lynch TJ, Bell DW, Sordella R, Gurubhagavatula S, Okimoto RA, et al (2004) Activating mutations in the epidermal growth factor receptor underlying responsiveness of non-small-cell lung cancer to gefitinib. N Engl J Med 350: 2129–2139. 15118073 10.1056/NEJMoa040938

[52] Mitsudomi T, Suzuki S, Yatabe Y, Nishio M, Kuwabara M, et al (1998) Clinical implications of p53 autoantibodies in the sera of patients with non-small-cell lung cancer. J Natl Cancer Inst 90: 1563–1568. 9790550 10.1093/jnci/90.20.1563

[53] Nagashio R, Sato Y, Jiang SX, Ryuge S, Kodera Y, et al (2008) Detection of tumor-specific autoantibodies in sera of patients with lung cancer. Lung Cancer 62: 364–373. 18485524 10.1016/j.lungcan.2008.03.026

[54] Puglisi F, Aprile G, Minisini AM, Barbone F, Cataldi P, et al (2001) Prognostic significance of Ape1/ref-1 subcellular localization in non-small cell lung carcinomas. Anticancer Res 21: 4041–4049. 11911289

[55] Chattopadhyay R, Das S, Maiti AK, Boldogh I, Xie J, et al (2008) Regulatory role of human AP-endonuclease (APE1/Ref-1) in YB-1-mediated activation of the multidrug resistance gene MDR1. Mol Cell Biol 28: 7066–7080. 18809583 10.1128/MCB.00244-08 PMC2593380

[56] Wu HH, Cheng YW, Chang JT, Wu TC, Liu WS, et al (2010) Subcellular localization of apurinic endonuclease 1 promotes lung tumor aggressiveness via NF-kappaB activation. Oncogene 29: 4330–4340. 20498636 10.1038/onc.2010.178

