# Supplementary figures and images for: Serum APE1 Autoantibodies: A Novel Potential Tumor Marker and Predictor of Chemotherapeutic Efficacy in Non-Small Cell Lung Cancer
Source: PLoS One. 2013 Mar 5;8(3):e58001. doi: 10.1371/journal.pone.0058001 (PMC3589448; doi:10.1371/journal.pone.0058001)

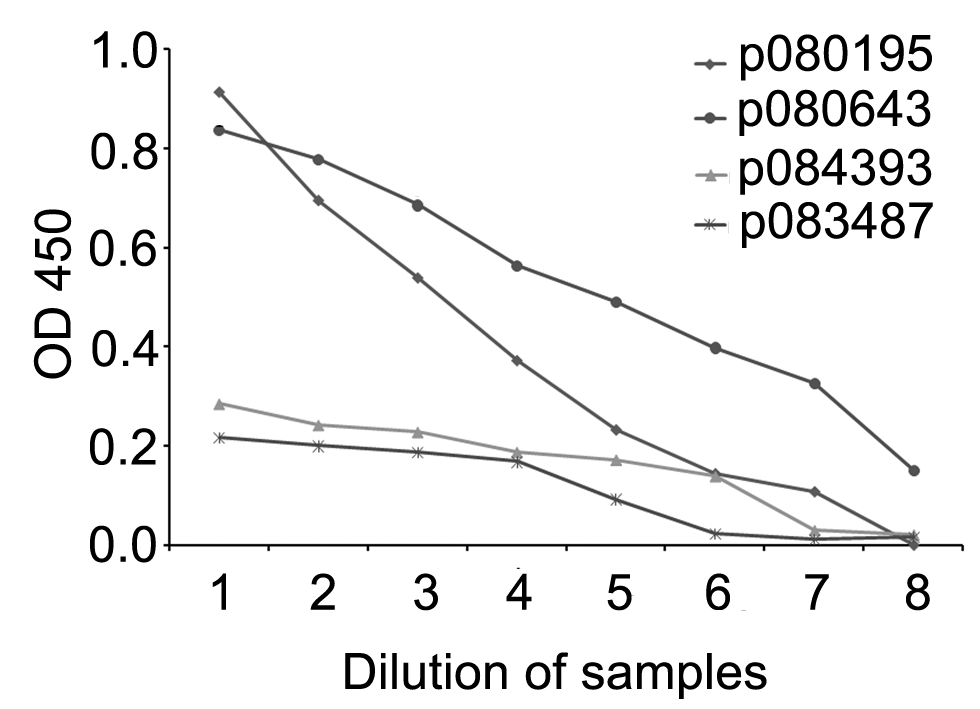

Supplement: Figure S1 — Optimal Serum dilution for ELISA technique. Optimal dilution was determined by titration technique. Dilution experiments showed that the APE1-AAbs titer differed from one patient to another. Two samples (p083487, p084393) from healthy controls displayed a low titer of APE1-AAbs while another two samples (p080195, p080643) from NSCLC patients had a high titer of APE1-AAbs. P080195 sample displayed a positive signal until diluted to 1∶1000, whereas p080643 showed a strong signal for a dilution of 1∶2000. These serum samples could be diluted 1∶1000 and still yield a positive signal. The optimal dilution of serum we selected was 1∶300. (TIF) [file pone.0058001.s001.tif]
