# Supplementary material for: Serum APE1 Autoantibodies: A Novel Potential Tumor Marker and Predictor of Chemotherapeutic Efficacy in Non-Small Cell Lung Cancer
Source: PLoS One. 2013 Mar 5;8(3):e58001. doi: 10.1371/journal.pone.0058001 (PMC3589448; doi:10.1371/journal.pone.0058001)
Supplement: Table S1 — Association between APE1 protein expression and serum APE1-AAbs in 42 NSCLC patients. *was correlation coefficient. The correlation of serum APE1-AAbs levels between nucleus expression group and ectopic expression group (cytoplasm expresssion and nucleus/cytoplasm coexpression) was statistically analyzed by Mann-Whitney. (DOCX) [file pone.0058001.s002.docx]

| **Patient No.** | **APE1 protein expression (score)** | **APE1-AAbs (OD)** | **Location** |
| --- | --- | --- | --- |
| Statistically analysis | Spearman *0.641 (<0.001) | | Mann-Whitney 0.610 |
| p070072 | 2 | 0.762 | nucleus/cytoplasm |
| p070140 | 2 | 0.949 | nucleus/cytoplasm |
| p070605 | 2 | 0.861 | nucleus/cytoplasm |
| p070716 | 1 | 0.421 | nucleus/cytoplasm |
| p070761 | 2 | 0.562 | nucleus/cytoplasm |
| p071427 | 2 | 0.857 | nucleus |
| p071794 | 1 | 0.458 | nucleus/cytoplasm |
| p072093 | 2 | 0.318 | nucleus/cytoplasm |
| p073471 | 2 | 0.633 | cytoplasm |
| p080004 | 1 | 0.321 | nucleus |
| p080038 | 0 | 0.091 | nucleus |
| p080178 | 1 | 0.306 | nucleus |
| p080468 | 1 | 0.499 | nucleus/cytoplasm |
| p080681 | 3 | 1.279 | nucleus/cytoplasm |
| p080706 | 3 | 0.726 | nucleus |
| p080799 | 3 | 0.954 | nucleus/cytoplasm |
| p081040 | 1 | 0.315 | nucleus/cytoplasm |
| p081042 | 1 | 0.623 | nucleus |
| p081131 | 1 | 0.830 | nucleus/cytoplasm |
| p081304 | 3 | 0.557 | nucleus |
| p081619 | 2 | 0.985 | nucleus/cytoplasm |
| p081646 | 2 | 0.762 | nucleus/cytoplasm |
| p081655 | 1 | 0.140 | nucleus/cytoplasm |
| p081863 | 2 | 0.957 | nucleus |
| p081937 | 3 | 1.2125 | nucleus/cytoplasm |
| p081977 | 2 | 1.311 | nucleus/cytoplasm |
| p082192 | 2 | 0.242 | nucleus/cytoplasm |
| p082247 | 2 | 0.346 | nucleus/cytoplasm |
| p082335 | 2 | 0.491 | nucleus/cytoplasm |
| p082444 | 2 | 0.358 | nucleus/cytoplasm |
| p082622 | 2 | 1.016 | nucleus |
| p082647 | 2 | 1.392 | nucleus/cytoplasm |
| p082765 | 0 | 0.321 | cytoplasm |
| p082979 | 2 | 0.352 | nucleus/cytoplasm |
| p082993 | 0 | 0.273 | nucleus |
| p083012 | 1 | 0.383 | cytoplasm |
| p083015 | 1 | 0.256 | nucleus |
| p083136 | 0 | 0.295 | cytoplasm |
| p083273 | 1 | 0.788 | nucleus/cytoplasm |
| p083509 | 2 | 0.567 | nucleus |
| p083527 | 3 | 2.292 | nucleus |
| p083589 | 2 | 1.014 | nucleus/cytoplasm |
