# Supplementary material for: Serum APE1 Autoantibodies: A Novel Potential Tumor Marker and Predictor of Chemotherapeutic Efficacy in Non-Small Cell Lung Cancer
Source: PLoS One. 2013 Mar 5;8(3):e58001. doi: 10.1371/journal.pone.0058001 (PMC3589448; doi:10.1371/journal.pone.0058001)
Supplement: Table S2 — Association between clinical characteristics and APE1 protein expression in NSCLC tissues. p values were calculated using chi-square test. (DOCX) [file pone.0058001.s003.docx]

| **Patients Characteristics** | **N (%)** | **APE1 protein expression** | | | | |
| --- | --- | --- | --- | --- | --- | --- |
|  |  | **Low expression (N)** | | **High expression (N)** | | ***p*** |
|  |  | 0 Score | 1 Score | 2 Score | 3 Score |  |
| Total | 42 | 4 | 12 | 20 | 6 |  |
| Age |  |  |  |  |  |  |
| <60 | 18 (42.86%) | 1 | 5 | 7 | 5 | 0.582 |
| ≥60 | 24 (57.14%) | 3 | 7 | 13 | 1 |  |
| Gender |  |  |  |  |  |  |
| Male | 32 (76.19%) | 4 | 8 | 18 | 2 | 0.887 |
| Female | 10 (23.81%) | 0 | 4 | 2 | 4 |  |
| Smoking status |  |  |  |  |  |  |
| Smoker | 26 (61.90%) | 4 | 6 | 16 | 0 | 0.95 |
| Non-smoker | 16 (38.10%) | 0 | 6 | 4 | 6 |  |
| Histological types |  |  |  |  |  |  |
| Adeno | 14 (33.33%) | 0 | 5 | 9 | 0 | 0.864 |
| Squamous | 26 (61.90%) | 4 | 6 | 10 | 6 |  |
| Others | 2 (4.76%) | 0 | 1 | 1 | 0 |  |
| TNM stage |  |  |  |  |  |  |
| Ⅰ+Ⅱ | 7 (16.67%) | 0 | 3 | 4 | 0 | 0.262 |
| Ⅲ | 17 (40.47%) | 2 | 2 | 8 | 5 |  |
| Ⅳ | 18 (42.86%) | 2 | 7 | 8 | 1 |  |
