# Supplementary material for: Serum APE1 Autoantibodies: A Novel Potential Tumor Marker and Predictor of Chemotherapeutic Efficacy in Non-Small Cell Lung Cancer
Source: PLoS One. 2013 Mar 5;8(3):e58001. doi: 10.1371/journal.pone.0058001 (PMC3589448; doi:10.1371/journal.pone.0058001)
Supplement: Table S3 — Association between serum APE1 antigen and serum APE1-AAbs in 137 NSCLC patients. *was correlation coefficient. (DOCX) [file pone.0058001.s004.docx]

| **Patients Characteristics** | **N (%)** | **Serum APE1 antigen** | ***p*** | **Spearman** |
| --- | --- | --- | --- | --- |
| Age |  |  |  |  |
| <60 | 64 (46.72%) | 0.72±0.45 | 0.848 |  |
| ≥60 | 73 (53.28%) | 0.71±0.41 |  |  |
| Gender |  |  |  |  |
| Male | 109 (79.56%) | 0.72±0.40 | 0.729 |  |
| Female | 28 (20.44%) | 0.75±0.46 |  |  |
| Smoking status |  |  |  |  |
| Smoker | 94 (68.61%) | 0.71±0.38 | 0.61 |  |
| Non-smoker | 43 (31.39%) | 0.76±0.48 |  |  |
| Histological types |  |  |  |  |
| Adeno | 57 (41.61%) | 0.72±0.41 | 0.963 |  |
| Squamous | 75 (57.74%) | 0.72±0.41 |  |  |
| Others | 5 (3.65%) | 0.92±0.54 |  |  |
| TNM stage |  |  |  |  |
| Ⅰ+Ⅱ | 16 (11.68%) | 0.52±0.37 | 0.293 |  |
| Ⅲ | 36 (26.28%) | 0.71±0.42 | 0.858 |  |
| Ⅳ | 85 (62.04%) | 0.77±0.41 | 0.071 |  |
| Total | 137 | 0.73±0.41 |  | 0.533*  *p*< 0.001 |
